# Sex-dependent effects of an early life treatment in rats that increases maternal care: vulnerability or resilience?

**DOI:** 10.3389/fnbeh.2014.00056

**Published:** 2014-02-25

**Authors:** Sílvia Fuentes, Núria Daviu, Humberto Gagliano, Pedro Garrido, Dóra Zelena, Nela Monasterio, Antonio Armario, Roser Nadal

**Affiliations:** ^1^Institut de Neurociències, Universitat Autònoma de BarcelonaBarcelona, Spain; ^2^Animal Physiology Unit, School of Biosciences, Universitat Autònoma de BarcelonaBarcelona, Spain; ^3^Institute of Experimental Medicine, Hungarian Academy of ScienceBudapest, Hungary; ^4^Psychobiology Unit, School of Psychology, Universitat Autònoma de BarcelonaBarcelona, Spain

**Keywords:** stress, maternal behavior, HPA axis, impulsivity, anxiety, novelty-seeking, sex

## Abstract

Early life stress (ELS) in rodents has profound long-term effects that are partially mediated by changes in maternal care. ELS not only induces “detrimental” effects in adulthood, increasing psychopathology, but also promotes resilience to further stressors. In Long-Evans rats, we evaluated a combination of two procedures as a model of ELS: restriction of bedding during the first post-natal days and exposure to a “substitute” mother. The maternal care of biological and “substitute” mothers was measured. The male and female offspring were evaluated during adulthood in several contexts. Anxiety was measured by the elevated plus-maze (EPM), acoustic startle response (ASR) and forced swim test (FST). In other group of animals, novelty-seeking was measured (activity in an inescapable novel environment, preference for novel environments and exploration of novel objects). Plasmatic ACTH and corticosterone in basal conditions and in response to stress were also measured. Cognitive impulsivity was assessed by a delay-discounting paradigm, and impulsive action, attention and compulsive-like behavior by a five choice serial reaction time task (5CSRTT). ELS decreased pup body weight and increased the care of the biological mother; however, the “substitute” mother did not exhibit overt maltreatment. A mixture of “detrimental” and “beneficial” effects was shown. In the 5CSRTT, attention was impaired in both genders, and in females, ELS increased compulsive-like behavior. Novel object exploration was only increased by ELS in males, but the preference for novel spaces decreased in both genders. Baseline anxiety (EPM and ASR) and recognition memory were not affected. Unexpectedly, ELS decreased the ACTH response to novelty and swim stress and increased active coping in the FST in both genders. Cognitive impulsivity was decreased only in females, but impulsive action was not affected. The enhancement in maternal care may “buffer” the effects of ELS in a context-dependent manner.

## Introduction

In rodents, early rearing conditions such as post-natal handling, environmental enrichment or stress have profound long-lasting consequences. Among these interventions, exposure to early life stress (ELS) has been repeatedly shown to promote detrimental behavioral and neurophysiological changes that last until adulthood. One of the most widely used models of ELS is periodic maternal separation that occurs for a few hours per day during the first post-natal days. Data indicate that maternal separation in rodents causes an important dysregulation of the hypothalamic-pituitary-adrenal (HPA) axis in adulthood, as reflected in enhanced corticotrophin-releasing factor (CRF) gene expression in several brain areas, including the paraventricular nucleus of the hypothalamus (Francis et al., 2002; Plotsky et al., 2005), and in the exacerbated responsiveness of ACTH and corticosterone to acute stressors (Liu et al., 2000; Francis et al., 2002; Huot et al., 2002). These changes in the HPA axis are associated with the behavioral indexes of hyper-anxiety (Huot et al., 2001).

However, the overall analysis of the literature indicates that the long-term effects of maternal separation are not consistent (Lehmann and Feldon, 2000), and a primary reason for these inconsistencies is related to maternal behavior. The importance of the infant-mother relationship for the correct development of the CNS and adult behavior is widely recognized (Fleming et al., 1999; Kaffman and Meaney, 2007). In fact, maternal separation and maternal care influence pups independently, but high levels of maternal care counteract the effect of the maternal separation (Macrì et al., 2008). Unfortunately, not all of the studies using maternal separation or other ELS procedures have measured their impact on maternal behavior across several time points through the day.

Considering the inconsistencies of the effect of maternal separation, in the present work, we used a novel model of ELS composed of a combination of two procedures. The first method was originally developed in rodents by the Baram laboratory and involves the restriction of nesting material to the biological mother. This manipulation induces stress in both the dams (Ivy et al., 2008) and the pups (Gilles et al., 1996), dysregulation of the HPA axis of the offspring in the short and long-term (Avishai-Eliner et al., 2001; Rice et al., 2008), passive behavior in the forced swim test (FST) and impairment in spatial learning and object recognition memory during adulthood (Brunson et al., 2005; Cui et al., 2006; Wang et al., 2011). In addition, this procedure induces several alterations of the hippocampal formation (Brunson et al., 2005; Ivy et al., 2010; Wang et al., 2011). The maternal behavior described in these studies is closer to neglect than to abuse. The second model uses the exposure of pups for a limited daily period to a “caregiver” (a non-biological or “substitute” mother that receives the pups without habituation time and with limited nesting material) that promotes maltreatment in the form of abusive behavior and induces persistent epigenetic changes in BDNF methylation (Roth and Sullivan, 2005; Roth et al., 2009).

Our specific aims were to characterize a new model of ELS in rats by evaluating in adulthood the effects on the following: (1) several cognitive/behavioral domains, such as impulsive action, attention and compulsive-like behavior (five choice serial reaction time task, 5CSRTT), impulsive choice (delay-discounting), anxiety (elevated plus-maze, EPM, and acoustic startle response, ASR), coping strategies in stressful situations (forced swim behavior), novelty-seeking measures (activity in a novel environment, preference for novel spaces, and preference for novel objects) and recognition memory; and (2) several markers of HPA axis functioning (ACTH and corticosterone plasma levels in basal situations and after different stressors). The inclusion of all of these measures in the same global experiment will allow us to define broad behavioral patterns in response to stress.

The present work aimed to study the consequences of exposure to ELS in both male and female offspring because vulnerability to stress is markedly gender-dependent (Becker et al., 2007; Goel and Bale, 2009; McEwen, 2010). The evaluation of maternal behavior was also included. No clear sign of maltreatment was detected in the “substitute” mother. Quite surprisingly, we observed that the procedure markedly promoted compensatory maternal behavior and resulted in not only negative consequences but also in a positive outcome in coping and emotional responsiveness during adulthood. Although several authors suggested that females may be more vulnerable to stress (Becker et al., 2007), our results indicate that both genders react to stress in a different way depending on the cognitive/behavioral domain evaluated. Overall, the present results also support the hypothesis that prior exposure to ELS may induce resilience (resistance) to further exposures to stress depending on several environmental factors (Gillespie et al., 2009; Stevens et al., 2009; Lyons et al., 2010; Russo et al., 2012; Southwick and Charney, 2012).

## Methodology

### Subjects

Long-Evans outbred rats were always housed in Makrolon transparent polycarbonate wire-topped cages with solid bottoms (26.5 × 42.5 × 18.5 cm, Ref. 1291 Eurostandard Type III H) containing sawdust bedding (Lignocel 3/4, Harlan) in a climate-controlled environment that was maintained at 20–21°C on a 12-h light–dark cycle (lights on 8:00 am). During gestation and lactation, the rats were fed with a high protein diet (Ref. 2918 Teklad Global 18% protein, Harlan) and after weaning, with a regular diet (Ref. 2014C Teklad Global 14% protein, Harlan). Behavioral studies were conducted during the light cycle except for initial overnight delay-discounting training. Rats were allowed *ad libitum* access to food, except during operant training (see below, delay-discounting and 5CSRTT). The rats always had free access to filtered tap-water.

All animal protocols were in accordance with the European Communities Council Directive 86/609/EEC and approved by the Ethics Committee for Human and Animal Research of the Universitat Autònoma de Barcelona and by the Catalan Government (Generalitat de Catalunya). A maximal effort was performed to minimize the number and suffering of animals.

### Early life-treatment

Mated pregnant dams arrived from Janvier (France) at GD 15. Regarding mating, each dam (approximately 8 weeks of age, primiparous) was paired with a different male (1 male/1 female) during the night. At the beginning of the experiment, we used 30 different dams. All of the dams were at the same vivarium and special care was taken to restrict access to the room. The dams were inspected twice daily for delivery (at 8:00 and at 15:00 h). The day of delivery was termed PND 0. The next day, the pups were counted and sexed and the litter culled to 12 if higher (maintaining, if possible, a sex ratio between 0.4 and 0.6) and weighed. Cross-fostering was never performed. At PND 1, the dams were divided at random into two groups: control (CTR) and ELS. The bedding was not changed until PND 8.

The ELS consisted of the combination of two different treatments: restriction of nesting material and exposure for 1 h/day to a “substitute” mother. The control dams remained undisturbed with the litter during the entire lactation period until weaning at PND 21. The ELS treatment lasted for 7 days, and then, all of the dams were returned to control conditions. The pups in each litter were weighed at PND 8.

The restriction of nesting material consisted of providing the dams with access to a limited amount of bedding (0.8 l instead of the 2.8 l that were given to CTR animals). Although the procedure was adapted from previous studies (Ivy et al., 2008), we did not use an aluminium plastic wire on the top of the bedding, and the dams had free access to the bedding, although the quality and size of the nest was compromised.

Spontaneous maternal behavior displayed by the biological mother during PND 1–7 was measured four times a day (8:30, 13:00, 16:00, and 20:30 h) following an adaptation of our previous studies (Dimitsantos et al., 2007; Llorente-Berzal et al., 2011; Fodor et al., 2012). Maternal behavior was also measured at PND 13 and PND 18 (i.e., 6 and 11 days after the treatment finished). The measures were recorded on-line by an observer that remained quietly in the room. Within each observation period, the behavior of each mother was scored 25 times spaced 3 min each (25 observations × 4 periods per day × 7 days = 700 observations/mother). The following behaviors were scored as present or absent: (1) mother licking-grooming (body + anogenital region) any pup, (2) mother nursing pups in an arched-back posture with rigid limbs (“high kyphosis”), (3) mother nursing in a “blanket” posture in which the mother just lies over the pups (“prone nursing”) or the mother limbs are rigid but maintains a low dorsal arch posture (“low/partial kyphosis”), (4) mother nursing in a “passive” posture (“supine nursing”) in which the mother lies on her back or side while the pups are nursing, and (5) mother “off” pups (no maternal contact). A more detailed description of the behavioral categories can be found elsewhere (Lonstein et al., 1998; Pryce et al., 2001; Champagne et al., 2003; Macrì et al., 2004).

As an additional measure of the “quality” of the maternal behavior, the variability (reliability) of licking-grooming and arched-back behaviors were calculated using a procedure adapted from Akers et al. (2008) and Tang et al. (2012). We removed the systematic decreasing trend due to the pass of time by fitting a straight line through the daily maternal behaviors of each dam and maintained the residuals for each of the 7 days. The variability index was the standard deviation of these daily residuals.

The behavior exhibited by the “substitute” mother was also measured. In fact, the “substitute” mother was another mother (different from the biological mother) that also participated in the experiment from the ELS group. The surrogate mother was introduced individually into an opaque white plastic cage opened at the top (33 × 33 × 37 cm) and filled with a limited amount of bedding (0.9 l). Immediately after, a litter (different from the own litter) was introduced inside the cage, and the behavior was video-recorded from the top of the cage for 60 min. Next, the pups were placed in an electric blanket (30°C) for 15 min after being returned to the vivarium. Each mother was exposed to the same litter during the first 7 post-natal days. A total of eight mothers were exposed at the same time in the same room. Later, the 30 first min of the behavior for each day was analyzed by the Observer software (Noldus, The Netherlands, version XT 11). Because expected to observe some type of maltreatment, the following behavioral patterns were measured (time and frequency): stepping on the pups, dropping them, dragging them, actively avoiding/pushing-away, and rough-handling (see Roth et al., 2009). “Normal” types of maternal behavior were also measured (as described above): licking-grooming, arched-back nursing, blanket-nursing, supine-nursing and “off” nest. This test occurred between the first and second measure of spontaneous maternal (biological) behavior.

A total of 253 pups was weaned (66 CTR-males, 69 ELS-males, 49 CTR-females, and 69 ELS-females). After weaning, the pups were housed by sex in groups of four (each one from a different mother) and kept undisturbed until PND 60. This day, the rats were housed in pairs and randomly assigned to five different experiments (with the four groups in each experiment: males and females, CTR and ELS). All of the rats were weighed at PND 27 and at PND 60. We named each experiment according to the primary behavior that was measured: anxiety, novelty-seeking, 5CSRTT and delay-discounting. An additional cohort was used for other experimental purposes, and the results are not reported here. For each experiment, in each of the four groups, only 1–2 pups from the same mother were used. Before initiating adult testing, the rats were handled for at least 3 days.

### General procedure at adulthood

In the anxiety experiment, the rats were tested as follows: (a) in the EPM at PND 64–65, (b) in the ASR test at PND 68–70 and (c) in the FST at PND 73–75. At PND 62–63, a blood sample was taken to measure HPA axis hormone basal levels, and after the FST, another blood sample was taken to measure HPA reactivity to stress. In the novelty-seeking experiment, the rats were tested as follows: (a) in the circular corridor at PND 71–72 as a measure of inescapable novelty-seeking test, (b) during PND 77–79 in the preference for a novel environment test and after the test another blood sample was taken to measure HPA reactivity to stress, and (c) during PND 104–107 in the exploration of novel objects and recognition memory test. In the 5CSRTT experiment, food restriction started at PND 63–65, and the last operant session was at PND 132–134. In the delay-discounting experiment, food restriction started at PND 63–65, and the last operant session was at PND 125–127. The animals were carried into the test rooms (adjacent to the “living” room) in their home-cages just before starting the test. Blood sampling was conducted in a different room. A summary of the performed procedures is shown in Table 1.

**Table 1:**
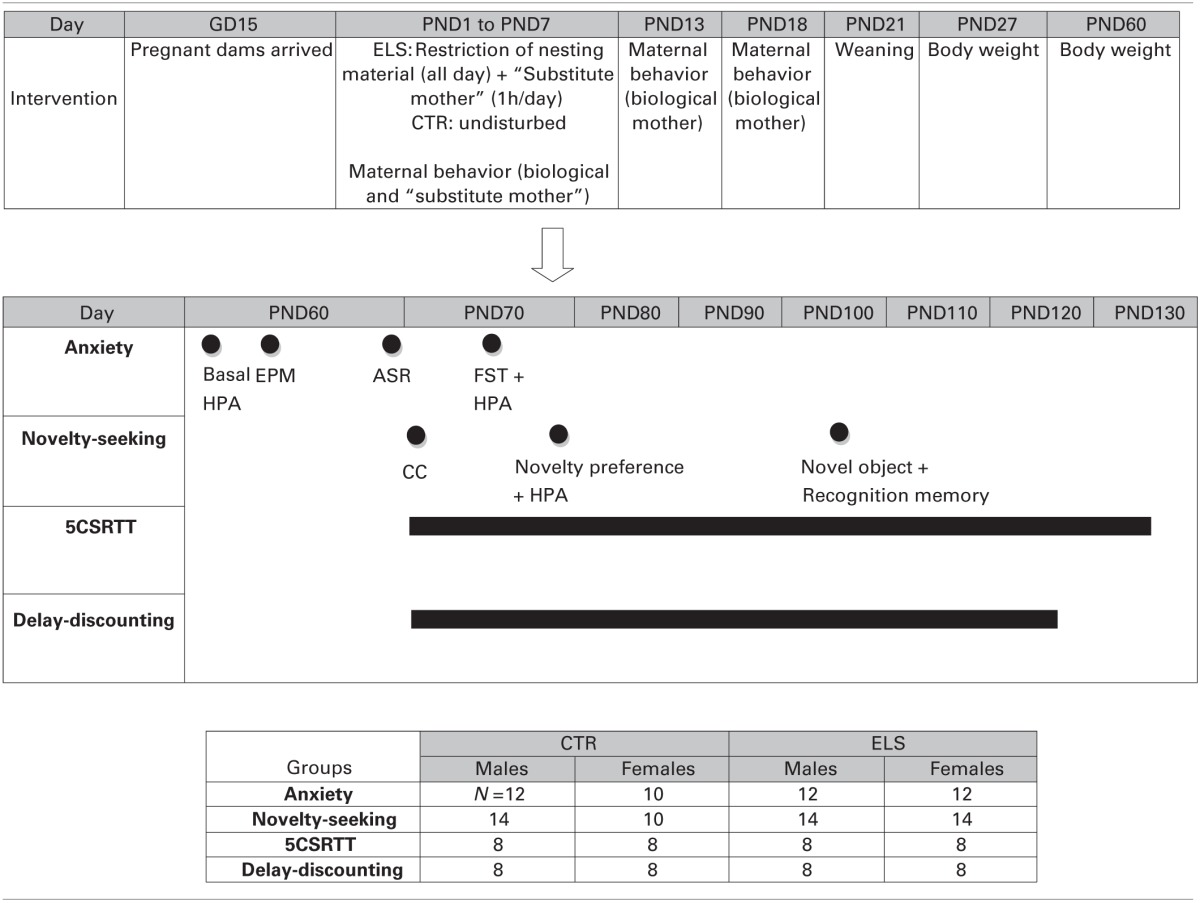
**General procedure of the different experiments**.

*Until PND 60 the general procedure was the same, and after that four different experiments were done. GD, gestational day; PND, post-natal day; ELS, early-life stress; CTR, control; HPA, hypothalamic-pituitary-adrenal axis activity; EPM, elevated-plus maze; ASR, acoustic startle response; FST, forced-swim test; CC, circular corridor; 5CSRTT, five-choice serial reaction time task*.

### Anxiety

The EPM adapted from Pellow and File (1986) consisted of four white wooden arms (formica) at right angles to each other and connected to a central square (10 cm^2^) to form the shape of a plus sign that was elevated 50 cm above the floor. Each arm was 46 cm long and 10 cm wide. Two of the opposite arms had high walls (enclosed arms, 43 cm high), whereas the other two were open arms that had a 0.7 cm ridge to provide an additional grip. The rat was placed facing a closed arm, and the subject was considered to be in a given arm when all of the paws were inside. One cage-mate was tested one day and the other cage-mate the next day. Thus, only one EPM was present in the room. The duration of the session was 5 min. The behavior was video-recorded from the top and analyzed by video-tracking by an observer blind to the treatment. The camera (Sony SSC-M388 CE, BW) was situated 150 cm above the center of the apparatus. A digital video recorder (JVC VR-716) sampled the position of the rat (8.3 samples/s) and was used to transfer the videos to a computer for video-tracking analysis using the center of gravity of the animal (Smart version 2.5.21, Panlab, S.L.U, Barcelona, Spain).

Two identical San Diego Instruments standard startle chambers (SR-LAB) were used to measure the ASR. The chambers had a clear Plexiglas tube (10.2 cm in diameter and 20.5 cm long), and a white light was ON during the entire session. Each chamber was placed inside a sound-attenuating box (“composite” type, 33 × 35 × 33 cm), and each box was located on an independent rigid counter. A constant 60 dB background white noise was provided by a fan placed inside each chamber. We followed a procedure adapted from Meloni and Davis (2004). After placing the animal inside the chamber, a 5 min period of acclimatization began where no stimulus (except the background) was administered. Startle responses were evoked by 50-ms bursts of white-noise at various intensities. First, five stimuli of 95 dB were used to habituate the animal (ISI: 30 s). Then, 100 startle stimuli were administered (20 stimuli at each of the intensities: 80, 85, 90, 95, and 100 dB, in a semirandom order, ISI: 30 s). The apparatus recorded the startle response during 100 ms after the onset of the pulse, with a sampling frequency of 1 kHz. The total duration of the session was 33 min. The chambers were calibrated daily with a standardization unit provided by San Diego Instruments. The intensity of the stimuli was checked using a Ref. 2240 Brüel & Kjaer sound level meter, with the microphone placed approximately at the same distance as the rat ear from the speaker. Between animals, the chambers were cleaned with a solution of 5% v/v ethanol diluted in tap water. The animals were weighed after the session because body weight affects the magnitude of the startle response (Young and Cook, 2004; Servatius et al., 2005). The startle response was defined as the highest voltage during the response window, that is, the “peak” of the response.

Regarding the FST exposure, the animals were allocated in transparent cylindrical plastic tanks (height 40 cm, internal diameter 19 cm) containing water (36°C) to a level of 22–24 cm (Armario et al., 1988; Dimitsantos et al., 2007). Four identical tanks were used, and four animals separated by black screens were exposed simultaneously. Water was always changed for each rat. During the 15 min test, (1) struggling/climbing, (2) immobility and (3) mild swimming were measured. Struggling was defined as diving, jumping or strongly moving all four limbs to break the surface of the water or scratching the walls. Immobility was defined as the animal remaining motionless to keep its head out of the water. Mild swimming was defined by subtracting from the total time of the test the time the animal spent struggling or immobile. The behavior was video-recorded from the front and later analyzed by an observer blind to the treatment with a stop-watch. As mentioned, a blood sample was taken after the test.

### Novelty-seeking

The corridor used to evaluate novelty-induced activity (Nadal et al., 2005) consisted of a white circular open field (80 cm diameter × 34 cm high) with a black floor and a white cylinder inside (52 × 34 cm) to form a corridor that was 14 cm wide that allowed for the movement of the rat. The rat was placed inside the corridor for 30 min. Two bulbs of 25 w were placed in the room to provide indirect light. The two animals from the same home-cage were tested simultaneously, and four corridors were placed in the same room. The behavior was video-recorded from the top, and the data were analyzed by video-tracking. After the session, the test area was cleaned with a solution of tap water containing ethanol (5% v/v).

For the novelty-preference test, a modified conditioned place-preference (CPP) apparatus was used (Nadal et al., 2005) that had two compartments (36.5 × 42 × 34.5 cm) with distinct visual and tactile cues: one compartment had stripped black and white walls with a wooden floor that was black and smooth, and the other compartment had white walls with black dots and a glass floor that was white, and, with guillotine doors that separated the different compartments. A procedure adapted from Molander et al. (2011) was used wherein each rat was confined to one of two compartments (counterbalanced across the rats) for two consecutive sessions (30 min each one). The next day, the separation between both compartments was removed, and the animals were allowed to freely explore both compartments for 15 min. The behavior was video-taped from the top of the cage and motor activity was recorded for day 1, and the time spent in each compartment during day 3 (new vs. familiar) was recorded by video-tracking. After the session, the test was cleaned with a solution of tap water containing ethanol (5% v/v). As mentioned before, a blood sample was taken after the test.

The open-field used for the novel object recognition task was similar to the circular corridor test without the cylinder inside. This test (Ennaceur, 2010) involved three phases, each separated by 24 h: (a) habituation to the open-field without objects (two sessions, 15 min each one); (b) exposure to two equal objects inside the open-field, “sample” trial (1 session, 10 min); and (c) exposure to two objects inside the same open-field, one familiar, and one novel “test” trial (1 session, 10 min). The “test” trial was used as a measure of recognition memory. For half of the rats, the two objects in the phase b were A-A and for the other half were B-B. During the “test” day (phase c), objects A-B were available for all of the rats. The objects were Lego constructions of different color/brightness and shape (“L” and “T” shape, both 10 cm high; one was 7 (wide) × 6.3 (long) cm and the other was 6.3 × 12.5 cm). The position of the novel object in the test session was counterbalanced across the groups. The objects were attached to the floor to avoid movement, and the position remained fixed across the two trials (“sample” and “test”), each at a distance of 10 cm from the wall in the middle of the open-field. Two bulbs of 25 w were placed in the room to provide indirect light. The two animals of the same home-cage were tested simultaneously, and four open-fields were placed in the same room. The animal was introduced inside the open-field facing the wall. The behavior was video-recorded from the top, and the time spent exploring each one of the two objects in phases b and c was recorded at blind by a stop-watch. Exploration was defined by sniffing (no less than approximately 1 cm from the object) or touching the objects. In the “test” trial, a discrimination index was calculated by the following formula: time spent exploring the novel object/(time exploring the novel object + time exploring the familiar object) * 100. After the session, the test area was cleaned with a solution of tap water containing ethanol (5% v/v).

### Five choice serial reaction time test

Two 5CSRTT chambers were used (Ref. MED-NP5M-D1, Med-Associates Inc., St. Albans VT, USA). The chambers (external dimensions: 31.8 × 25.4 × 34.3 cm, “extra-tall”) had two walls made of aluminium and two made of clear Plexiglas. One of the walls was curved and contained five holes (2.5 × 2.5 cm) that were 2.4 cm deep and 2.4 cm apart, placed 6 cm from the floor. Each hole had an IR detector (1 cm from aperture) for nose-poke and a light inside (LED, 6.4 mm in diameter). The chambers had a stainless steel grid floor and a waste pan. The pellet dispenser was connected to a food magazine (5.4 × 5 cm) with a light inside (1 cm in diameter) and a head entry detector (IR) that was placed in the opposite wall from the holes. Each hole was placed 33 cm from the food magazine. The house-light (28 V DC, 100 mA) was placed 30 cm from the floor, near the roof and above the food magazine. The chambers were placed inside a PVC sound-attenuating cubicle (63.5 × 40 × 60.5 cm) with a fan that provided a background noise of 60 db. The chambers were controlled by the Med-Associates software.

The animals were food deprived by providing them with a restricted amount of food (18 g/day for males, and 15 g/day for females) to maintain specific growth across days. Pellets from Bio-Serv (45 mg, Ref. F0021) were used as a reinforcer. The animals were always fed at least 1 h after the end of the session. The rats were run for 5 days/week. The chambers were carefully cleaned between rats with a solution containing soap. We followed the general procedure described in Bari et al. (2008). The session took place in a darkened room. Each session started with the illumination of the house-light and the magazine-light and the administration of a pellet (without requiring a nose-poke). When the rat collected the pellet inside the food magazine, the magazine-light was extinguished and an ITI was started. Responding to the food magazine during the ITI restarted it. After the ITI, a brief light inside one of the five holes was provided. If the animal performed a correct response (a nose-poke in the hole where the light was given) during a limited-hold time, the animal received a pellet, and the magazine-light was again illuminated. When the animal made a correct response, the stimulus-light (hole-light) was extinguished. If the animals failed to respond during this limited-hold period (omission) or made an incorrect response (a nose-poke in another non-illuminated hole), the program entered in a TO (always 5 s) during which the rat did not receive a reward and the house-light was OFF. Responses in any hole before the light (during the ITI) also produced a TO. The responses in the holes made during the TO also re-started the TO. Responding to the food magazine during the TO initiated a trial. After the TO, the house-light and the magazine-light were again illuminated, and the rat had to nose-poke inside the food magazine to start a new trial. The stimulus-light (inside the holes) was presented the same number of times in each hole during the complete session in a random order. Each daily session consisted of 90-100 trials (trials finished, which is the sum of correct responses + incorrect responses + omissions) or 30 min of testing, whichever was first achieved. At the end of each session, all of the lights were extinguished.

The animals were trained until they arrived at the following target parameters: 1 s of stimulus (stimulus-light) duration, 5 s of ITI and 5 s of limited-hold (what is called “phase 7” in Bari et al., 2008, see Table 2). The variables measured included the following: correct responses, incorrect responses (errors of “commission”), premature responses (anticipatory responses made during the ITI, as a measure of motor impulsivity), responses during the TO (as another measure of inhibitory control), perseverative responses (as a measure of compulsivity: an additional response performed in any hole after a correct response before collecting the reward, these responses did not produce TO) and errors of omission (when no response was made during the limited-hold period, measure that reflects motivational/motor deficits). The level of accuracy (as a measure of sustained and spatially divided visual attention) was calculated by the following formula: the number of correct responses/(the number of correct + the number of incorrect responses,) expressed as a percentage. The other measures taken included the following: the number of trials finished, duration of the session, latency of the first response (correct or incorrect, always after the beginning of the first stimulus-light), latency of the correct responses (from the beginning of the light to the entry inside the hole, measured by the interruption of the IR light, which is a measure of the speed of processing), latency of the incorrect responses and latency to collect the reward (time elapsed from the correct response to the head entry inside the food magazine, measured by the interruption of the IR light, which is a measure of the motivation/decision-time).

**Table 2:** **Training phases in the 5CSRTT**.

**Training phase**	**Stimulus duration (s)**	**ITI (s)**	**Limited hold (s)**	**Criterion to move to next phase**
1	30	2	30	≥30 Correct trials
2	20	2	20	≥30 Correct trials
3	10	5	10	≥50 Correct trials
4	5	5	5	≥50 Correct trials
				>80% Accuracy
5	2.5	5	5	≥50 Correct trials
				>80% Accuracy
				<20% Omissions
6	1.25	5	5	≥50 Correct trials
				>80% Accuracy
				<20% Omissions
7	1	5	5	

*ITI, Inter-trial-interval. Adapted from Bari et al. (2008)*.

### Delay-discounting

The delay-discounting task was conducted in eight standard Skinner boxes (Panlab S.L.U., LE1005, Barcelona, Spain). Each chamber (25 × 25 × 25 cm) had a clear Plexiglas door and black aluminium sidewalls. The floor was composed of 19 stainless steel rods (3 mm in diameter), spaced 1 cm from center to center. A house-light (4-cm diameter 2.4-W, 24-V) was placed in the right wall at 22 cm from the floor. In the left wall, two metal retractable response levers (6 cm above the floor) were placed on either side of a food magazine (3.5 × 3.5 cm). Another light was placed above each lever and above the food magazine (three lights in total). The software (Packwin 2.00.2, Panlab S.L.U.) controlled the administration of the different stimuli and recorded the data. The chambers were inside a metallic sound-attenuating box (67 × 53 × 55 cm) provided with a fan that helps to mitigate strange sounds.

The animals were initially food deprived until 85% of their free body-weight was reached and were then given 15–18 g of food each day to assure normal growth throughout the sessions. Bio-Serv pellets (45 mg, Ref F0021, Frenchtown, NJ, USA) were used as a reinforcer. The animals were always fed at least 1 h after the end of the session, and the rats were run 5 days/week. The chambers were carefully cleaned with a solution containing soap between rats.

The procedure involved three phases of pre-training previously to the delay-discounting test. In the first phase, overnight sessions were conducted with the house-light always ON and the two levers accessible (not retracted) and active to provide 1 pellet after 1 lever press (FR1) until 100 reinforcers were obtained. In the second phase, the session started with all of the lights OFF, and the two levers were retracted for a period of 3 min. Next, a non-contingent pellet was given at the same time that the house-light and the magazine-light was ON. When the rat nose-poked inside the food magazine, the magazine-light switch to OFF, and one of the two levers was then accessible. If the rat pressed the lever within a limited-hold of 30 s, the lever was retracted and 1 pellet was given. If the rat failed to respond (“omission”), the lever was also retracted but no pellet was given, and the house-light was switched OFF. Then, the next trial started with the illumination of the house-light and the magazine-light. The session ended when the animal performed 100 trials, and the session was repeated the next day until at least 60 reinforcers were obtained, and both levers were pressed at least 30 times. In the third phase of pre-training, the procedure was basically the same, but we introduced a limited-time of 10 s between the illumination of the house-light and the magazine-light and the nose-poke, the limited-hold period to press the lever was decreased from 30 to 10 s, and the session finished after 60 trials or 90 min. There was no delay with any of the 2 levers in this pre-training.

After pre-training, the proper delay-discounting procedure started. A between-sessions procedure was used, adapted from Winstanley et al. (2004) and Cardona et al. (2006). One lever (A, the “immediate”) produced a single pellet as a reinforcement after 1 lever press, and the other lever (B, the “delayed”) produced 4 pellets. The position of levers A and B (right/left) was counterbalanced across rats. During the weeks of testing, the delay of reinforcement presentation after the lever press was changed. During the first week of testing (5 days), the delay was 0 s; during the second, it was 10 s; during the third, it was 20 s; during the fourth, it was 40 s; and during the last week, it was 0 s to ensure that the training and/or the passage of time did not modify the preference for the B lever when there was no delay (4 pellets). Lever A never had a delay. Each session consisted of 60 trials or 90 min total. The basic procedure was the same as before, except now half of the trials were “forced” and only 1 lever was accessible (15 times lever A and 15 times lever B), and the other half of the trials were “free” (“choice” or “decision” trials), where both levers were presented and the animal had to choose which to press. Each session was divided into five identical blocks of 12 trials in which the first six trials were of forced choice, and the last six were free choice trials. The start of each trial was signaled by turning the house-light and the magazine-light ON. A nose-poke response was required in the food magazine to produce the lever presentation. A failure to make a nose-poke in the food magazine or subsequently to depress the lever within 10 s (“omission”) terminated the trial. Thus, in each of the “free” trials, the subject had three possibilities: press lever A, press lever B or do not press the levers (omissions). When one of the two levers was pressed, the house-light was turned OFF and both levers were retracted. The food reward was then delivered, accompanied by the onset of the magazine-light, either immediately or after a delay (that was no signaled). When the food was collected, the magazine-light was switched OFF, and the chamber returned to the ITI state.

### Blood sampling and radioimmunoassay

During the last day of handling, the rats were subjected to a tail nick procedure to habituate them to the blood sampling procedure when needed. The tail nick consisted of gently wrapping the animals with a cloth, making a 2-mm incision at the end of one of the tail veins and then massaging the tail while collecting 300 μl of blood into ice-cold EDTA capillary tubes (Sarsted, Granollers, Spain) within 2 min. After centrifugation at 4°C, the plasma was stored at −20°C. In all of the experiments, cage-mates were processed simultaneously, including blood sampling (two experimenters were sampling at the same time and a third was gently holding the two rats). Tail-nicking is a procedure that is extensively used because low resting levels of hormones are obtained (Belda et al., 2004; Vahl et al., 2005). Animals were always tested in a different room from the animal room and blood sampling room.

Plasma ACTH and corticosterone levels were determined by a double-antibody radioimmunoassay (RIA) following our general procedures. Briefly, ACTH RIA used ^125^I-ACTH (PerkinElmer Life Science, Boston, USA) as the tracer, rat synthetic ACTH 1–39 (Sigma, Barcelona, Spain) as the standard and an antibody raised against rat ACTH (rb7) kindly provided by Dr. W.C. England (Department of Surgery, University of Minnesota, Minneapolis, USA). The characteristics of the antibody have been previously described (Engeland et al., 1989), and we followed a non-equilibrium procedure. Corticosterone RIA used ^125^I-corticosterone-carboximethyloxime-tyrosine-methylester (ICN-Biolink 2000, Barcelona, Spain), synthetic corticosterone (Sigma, Barcelona, Spain) as the standard and an antibody raised in rabbits against corticosterone–carboximethyloxime-BSA kindly provided by Dr. G. Makara (Institute of Experimental Medicine, Budapest, Hungary). The characteristics of the antibody and the basic RIA procedure have been previously described (Zelena et al., 2003). All of the samples that were statistically compared were run in the same assay to avoid inter-assay variability. The intra-assay coefficient of variation was 5.1% for ACTH and 7.6 % for corticosterone. The sensitivity of the assays was 25 pg/ml for ACTH and 2 ng/ml for corticosterone.

### Statistical analysis

Data were analyzed by the Statistical Program for Social Sciences, SPSS (version 18). A repeated-measures analysis of variance (GLM) was used with two between-subjects factors: GENDER (two levels, males and females) and early treatment (two levels, CTR and ELS). If needed, a within-subject factor was included (TIME). To achieve homogeneity of variances, if needed, log-transformations were performed. If log-transformations were not useful (because the data had several zeroes or because after the transformation the data did not achieve homogeneity of variances), a generalized linear model or GzLM (McCulloch et al., 2008) instead of a GLM was performed. Specific statistical analyses are explained in the corresponding section. Data are given as the mean ± s.e.m. Statistical significance was set at *p* < 0.05.

## Results

### General data: ELS decreased body weight until early adulthood

**Figure 1:**
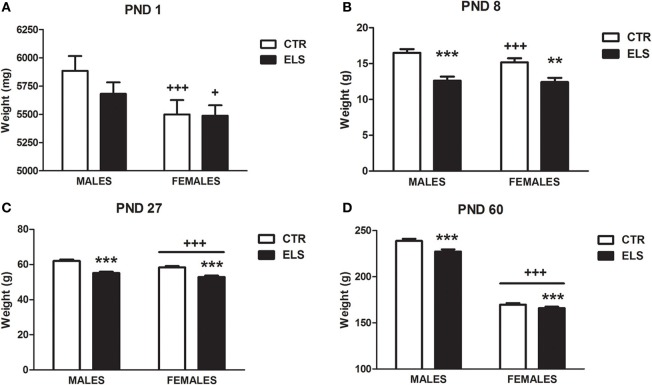
**Body weight in g (X ± s.e.m.) in males and in females at post-natal day (PND) 1 (A, before the treatment), PND 8 (B, after the treatment), PND 27 (C) and at PND 60 (D), in control (CTR) and early-life stress (ELS) rats**. ^**^p < 0.01, ^***^p < 0.001 vs. CTR, ^+^*p* < 0.05, ^+++^*p* < 0.001 vs. males. Body weight at PND 1 and PND 8 was analyzed together and the GENDER × TIME × ELS effect was statistically significant. Separate analysis were done for PND 27 and PND 60, and GENDER and ELS effects were statistically significant, but not the interaction.

One dam (CTR group) was excluded from the study because it exhibited cannibalism, leaving a total of 29 dams. The births were scattered across 3 days. The litter size and sex ratio was not different between groups after they were culled (data not shown). Regarding body weight previous to weaning, a repeated-measures analysis of variance was performed using “dam” as the subject (all of the pups of the same dam were weighed together, separated by gender), two within-subjects factors (GENDER and TIME, both two levels) and one between-subjects factor (ELS, two levels). Statistical analysis showed that GENDER [*F*_(1, 26)_ = 546.95, *p* < 0.001], TIME [*F*_(1, 26)_ = 40.62, *p* < 0.001], GENDER × ELS [*F*_(1, 26)_ = 18.81, *p* < 0.001], TIME × ELS [*F*_(1, 26)_ = 15.19, *p* = 0.001], GENDER × TIME [*F*_(1, 26)_ = 9.38, *p* < 0.01], GENDER × TIME × ELS [*F*_(1, 26)_ = 9.09, *p* < 0.01] were significant. To analyse the triple interaction, separate analyses were conducted for males and females. Figure 1A shows that at PND 1, prior to the treatment, the pup body weight was not affected by the group; however, at PND 8 (Figure 1B), the pups exposed to ELS weighed less, both in males and in females.

After weaning, the body weight was studied using the “pup” as the subject (each pup was weighed individually) and three between-subjects factors (ELS and GENDER, both with two levels, and COHORT, with five levels). The results showed (Figures 1C,D) that the decrease in body weight induced by ELS was maintained at PND 27 and at PND 60 in both males and females regardless of the cohort (experiment). At PND 27, statistical analysis indicated that GENDER [*F*_(1, 230)_ = 13.31, *p* < 0.001] and ELS [*F*_(1, 230)_ = 56.42, *p* < 0.001] were the only statistically significant factors. The same results were obtained for PND 60 [GENDER: *F*_(1, 230)_ = 958.20, *p* < 0.001; ELS: *F*_(1, 230)_ = 12.63, *p* < 0.001]. Additional body weight measures beyond PND 60 were only taken in several experiments. In the “anxiety cohort,” body weight was measured after the ASR session (PND 68–70), and the results (data not shown) indicated that the ELS effect was dissipated [GENDER: *F*_(1, 42)_ = 250.84, *p* < 0.001, ELS and ELS × GENDER: NS].

### Maternal behavior: ELS increased maternal behavior in the biological mother without any sign of maltreatment by the “substitute” mother

For each maternal behavior demonstrated by the biological mother, episodes during each of the first post-natal 7 days were calculated (from the 4 measures taken for each day). In each case, a repeated-measures analysis of variance was performed with DAY (seven levels) as the within-subject factor and ELS (two levels: CTR and ELS) as the between-subject factor.

**Figure 2:**
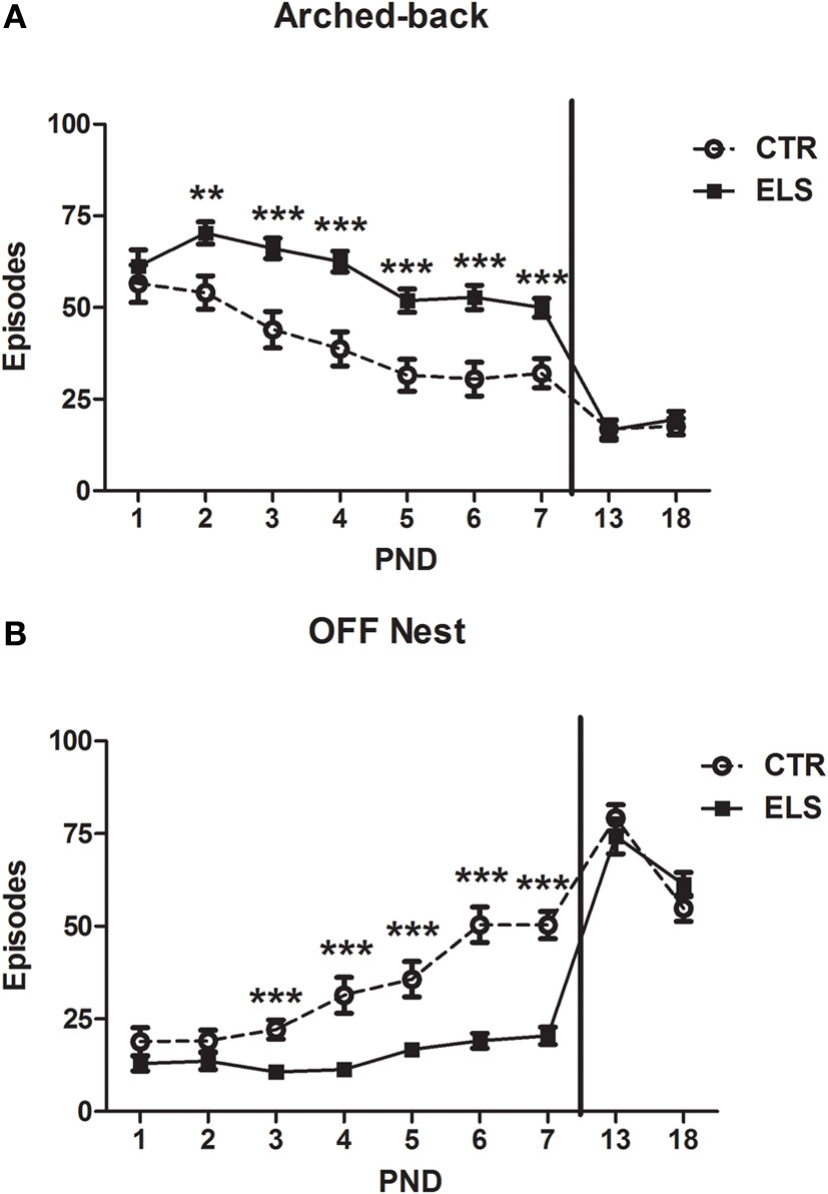
**Number of episodes (X ± s.e.m.) of arched-back (A) and “off” nest (B) behaviors during the first seven post-natal days (PND) and in PND 13 and PND 18, for control (CTR) and early-life stress (ELS) dams**. For each day all the measures realized are collapsed. ^**^p < 0.01 and ^***^p < 0.001 vs. CTR. In both cases, the DAY × ELS effect was statistically significant.

The arched-back nursing results are shown in Figure 2A. The repeated-measures analysis of variance showed that DAY [*F*_(6, 162)_ = 20.55, *p* < 0.001], ELS [*F*_(1, 27)_ = 25.45, *p* < 0.001] and DAY × ELS [*F*_(6, 162)_ = 2.93, *p* < 0.05] were statistically significant. The decomposition of the interaction showed that ELS mothers spent more time in the arched-back behavior during PND 2–7 (between *p* < 0.01 and *p* < 0.001). After the end of the treatment, in PND 13 and PND 18, the differences between the groups dissipated. The arched-back behavior decreased across time in both groups. Although the arched-back behavior increased with ELS treatment, the behavior of ELS mothers appeared to have a different pattern than CTR dams. When the ELS dam was in the typical arched-back posture, instead of having all of the pups below her, the nest was disaggregated; therefore, the arched-back behavior was only addressed specifically to several of the pups. The number of pups out of the nest when the dam was performing an arched-back was not systematically measured in each observation, but in some days, if at least one pup was out of nest, this behavior was recorded. For example, at PND 3, no CTR dam had any pup out of the nest; however, 12 out of 16 ELS dams had at least one pup out of the nest [χ^2^(1) = 17.5, *p* < 0.001].

The number of episodes for which the dam was “off” the nest is shown in Figure 2B. Statistical analysis showed that DAY [*F*_(6, 162)_ = 23.39, *p* < 0.001], ELS [*F*_(1, 27)_ = 36.88, *p* < 0.001] and DAY × ELS [*F*_(6, 162)_ = 7.33, *p* < 0.001] were statistically significant. The decomposition of the interaction showed that ELS mothers spent less time “off” the nest from PND 3 to 7 (between *p* < 0.01 and *p* < 0.001). After the end of the treatment, from PND 13 and PND 18, the differences between the groups dissipated. In general, the “off nest” behavior increased over time.

**Figure 3:**
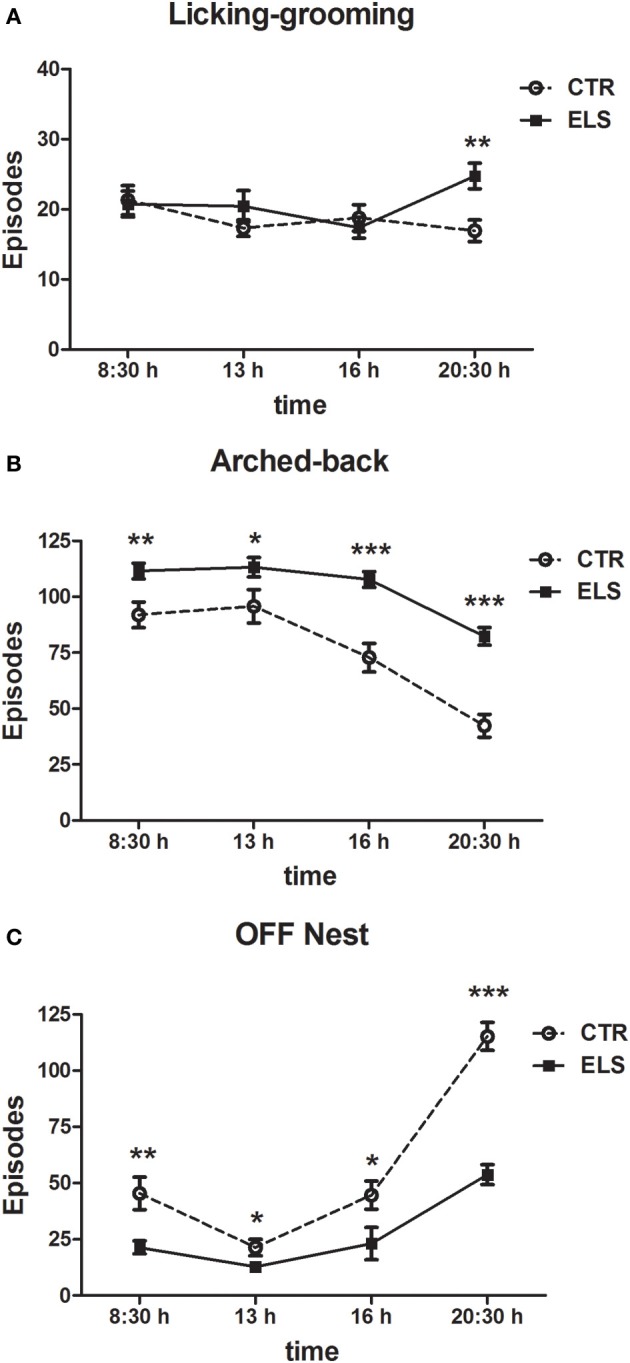
**Number of episodes (X ± s.e.m.) of licking-grooming (A), arched-back (B) and off-nest (C) behaviors during the first seven post-natal days in four different times of day, for control (CTR) and early-life stress (ELS) dams**. ^*^p < 0.05, ^**^p < 0.01, and ^***^p < 0.001 vs. CTR. In both cases, the HOUR × ELS effect was statistically significant.

Maternal behavior was also analyzed for each time of the day that was analyzed, including the 7 post-natal days (08:30, 13:00, 16:00, and 20:30 h). In this case, the within-subject factor was HOUR instead of DAY. The results regarding licking-grooming behavior are shown in Figure 3A, and the statistical results indicated that HOUR and ELS were not statistically significant, but the interaction HOUR × ELS was significant [*F*_(3, 81)_ = 3.54, *p* < 0.05]. The decomposition of the interaction showed that at night, ELS mothers licked-groomed more their pups (*p* < 0.01). Regarding arched-back behavior (Figure 3B), statistical analysis showed that all of the factors were significant: HOUR [*F*_(3, 81)_ = 57.86, *p* < 0.001], ELS [*F*_(1, 27)_ = 25.45, *p* < 0.001] and HOUR × ELS [*F*_(3, 81)_ = 4.82, *p* < 0.01]. The decomposition of the interaction indicated that ELS dams presented more arched-back behavior than CTR dams at all of the times analyzed (between *p* < 0.05 and *p* < 0.001); the differences were more apparent at 16 h and at 20:30 h. The results regarding “off” nest behavior are shown in Figure 3C, and the statistical analysis indicated that all of the factors were significant: HOUR [*F*_(3, 81)_ = 80.80, *p* < 0.001], ELS [*F*_(1, 27)_ = 36.88, *p* < 0.001] and HOUR × ELS [*F*_(3, 81)_ = 12.22, *p* < 0.001]. The decomposition of the interaction showed that ELS decreased “off” nest behavior at all of the times analyzed (between *p* < 0.05 and *p* < 0.001), but the differences were more apparent at night.

**Figure 4:**
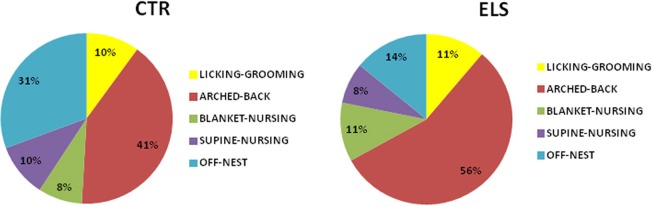
**Distribution of different types of maternal behavior in control (CTR) and early-life stress (ELS) dams during the first seven post-natal days**.

Regarding the other maternal measures (blanket-nursing and supine-nursing), no differences between-groups were detected (data not shown). Total nursing behavior across the first 7 post-natal days (including arched-back, supine, and blanket-nursing) was also increased in ELS vs. CTR dams [*t*_(27)_ = 6.46; *p* < 0.001]. The groups did not differ in maternal care “variability” (licking-grooming: 3.52 ± 0.37 in CTR animals vs. 4.12 ± 0.32 in ELS animals, NS; arched-back: 9.15 ± 0.82 in CTR animals vs. 9.99 ± 1.03 in ELS animals, NS). In Figure 4, a summary of the pattern of maternal behaviors exhibited by each group is presented.

**Figure 5:**
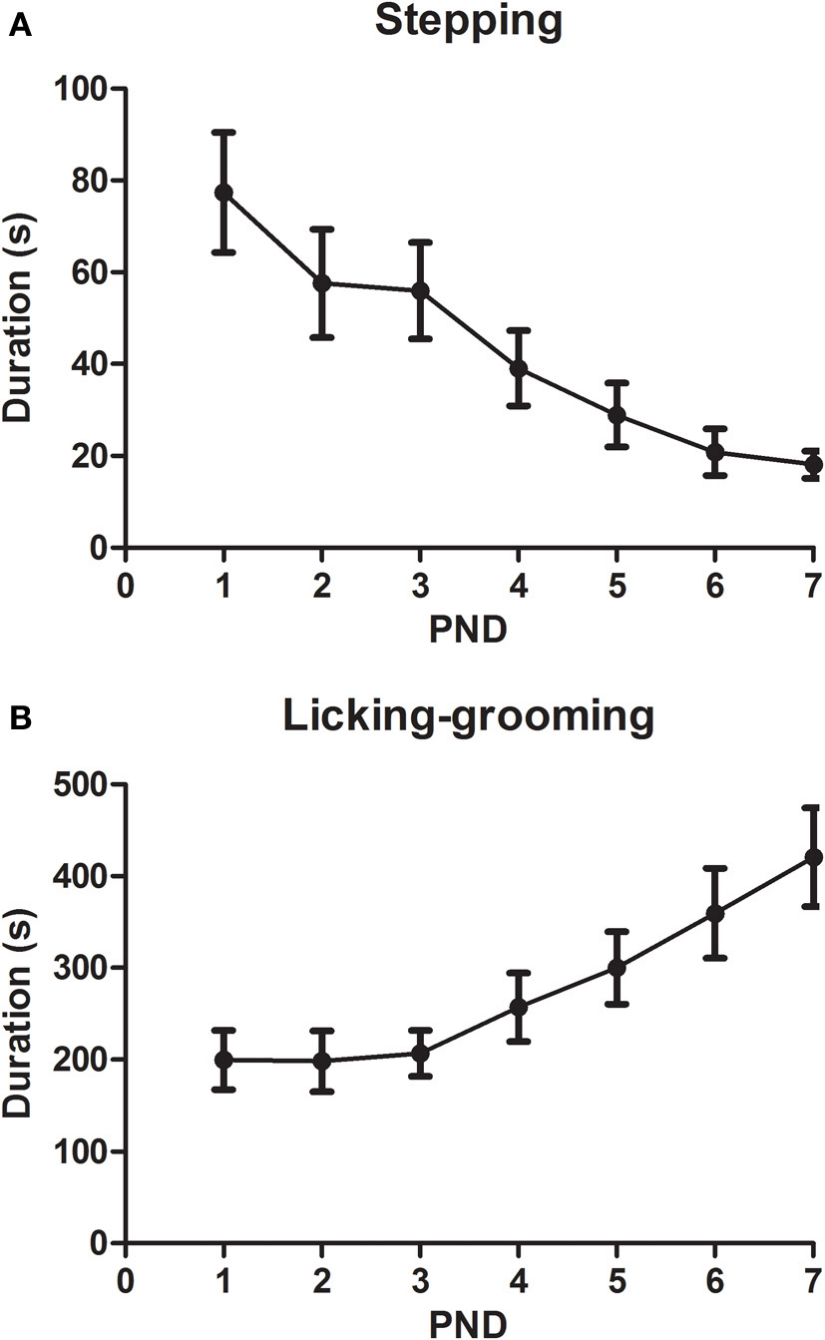
**Duration (X ± s.e.m.) of stepping (A) and licking-grooming (B) behavior realized by the substitute mother (ELS group) during the first seven post-natal days (PND) in the first 30 min of exposure**. In both cases, the DAY effect was statistically significant.

When the pups were exposed to the “substitute” mother (Figure 5), the only sign of putative maltreatment was “stepping” behavior. This behavior decreased during the first 7 post-natal days [DAY: *F*_(6, 90)_ = 6.11, *p* < 0.001] following a linear trend [*F*_(1, 15)_ = 22.81, *p* < 0.001]. The “normal” active maternal behavior, licking-grooming, was also observed in those dams, and this measure increased across days [DAY: *F*_(6, 90)_ = 6.6, *p* < 0.001] following a linear trend [*F*_(1, 15)_ = 15.26, *p* = 0.001].

In conclusion, ELS induced a transient increase in active maternal behavior that was especially evident at night in the biological mothers. When the treatment finished, the effect dissipated. The biological maternal behavior variability was not affected by the treatment. “Substitute” mothers did not present any sign of maltreatment toward the pups, except for the “stepping” behavior that decreased along days.

### Anxiety: ELS did not modify EPM or ASR behavior, but increased active behavior in the FST

**Figure 6:**
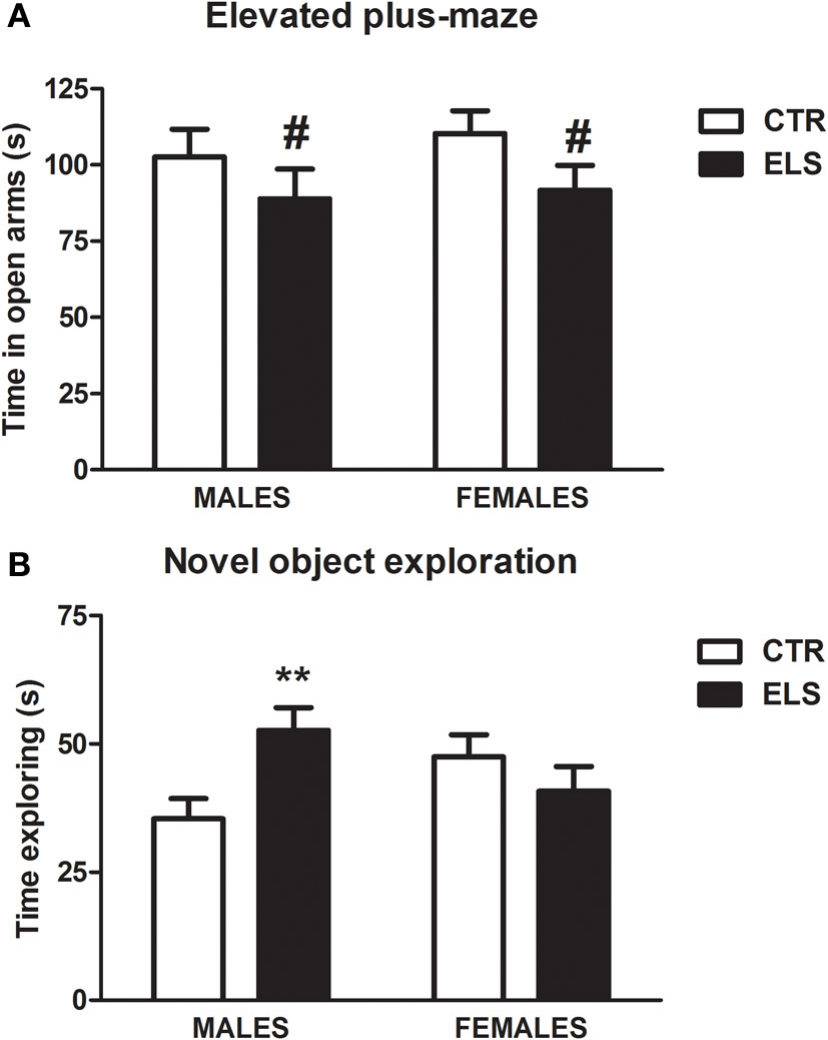
**(A)** Time in sec (X ± s.e.m.) in the open arms of the elevated plus-maze, in males and females for control (CTR) and early-life stress (ELS) rats. # = 0.066 vs. CTR. **(B)** Time in sec (X ± s.e.m) exploring a novel object, in males and females for control (CTR) and early-life stress (ELS) rats. ^**^p < 0.01 vs. CTR. For **(B)**, the GENDER × ELS effect was statistically significant.

ELS tended to decrease the time spent in open arms in the EPM (Figure 6A, *p* = 0.066), whereas GENDER and GENDER × ELS were both NS. General motor activity was not affected by ELS. In several measures reflecting general activity (data not shown), the females were more active than males [GENDER factor in closed arm distance: *F*_(1, 41)_ = 18.03, *p* < 0.001; in closed arms entries: *F*_(1, 41)_ = 6.38, *p* < 0.05, in open arms distance: *F*_(1, 41)_ = 5.10, *p* < 0.05, in center distance: *F*_(1, 41)_ = 6.73, *p* = 0.05, in center entries: *F*_(1, 41)_ = 5.86, *p* < 0.05]. Regarding ASR data (see Table 3A), analysis showed the expected effect of PULSE INTENSITY [*F*_(4, 168)_ = 35.24, *p* < 0.001], but all of the other factors and interactions were NS. The inclusion of body weight as a covariate in the analysis did not modify the results.

**Table 3:** **Different behavioral and endocrine responses observed in male and female rats exposed or not to an early-life stress (CTR or ELS)**.

	**Groups**	**Males**	**Females**
**A. ACOUSTIC STARTLE REFLEX**			
CTR	Vmax_80	16.8 ± 1.7	18.7 ± 1.3
	Vmax_85	19.6 ± 1.7	20.5 ± 2.0
	Vmax_90	20.8 ± 2.4	20.4 ± 1.8
	Vmax_95	20.9 ± 1.1	28.3 ± 4.6
	Vmax_100	34.5 ± 3.7	33.0 ± 6.0
ELS	Vmax_80	19.3 ± 1.9	20.4 ± 2.5
	Vmax_85	19.4 ± 1.4	16.5 ± 1.8
	Vmax_90	19.7 ± 2.1	18.3 ± 1.7
	Vmax_95	23.6 ± 2.3	21.5 ± 2.1
	Vmax_100	44.0 ± 6.5	33.7 ± 5.7
**B. DISTANCE TRAVELED IN NOVEL ENVIRONMENTS**			
CTR	Circular corridor	10250.3 ± 436.0	11278.9 ± 597.8
	Novelty preference	4318.7 ± 324.1	5799.5 ± 774.9
ELS	Circular corridor	9576.1 ± 417.2	12062.9 ± 866.2
	Novelty preference	4213.9 ± 290.6	5784.8 ± 743.6
**C. OBJECT RECOGNITION MEMORY**			
CTR	Object F	20.2 ± 2.3	17.6 ± 2,9
	Object N	24.4 ± 2.5	23.0 ± 3.2
	Discrimination	55.4 ± 3.8	56.6 ± 4.5
ELS	Object F	17.2 ± 3.2	20.2 ± 2.6
	Object N	26.5 ± 4.0	24.4 ± 2.1
	Discrimination	61.8 ± 2.6	56.3 ± 3.7
**D. BASAL HORMONE LEVELS**			
CTR	ACTH	70.3 ± 7.3	158.0 ± 19.6
	CORT	6.3 ± 3.3	170.5 ± 54.9
ELS	ACTH	109.7 ± 36.7	208.6 ± 57.4
	CORT	41.8 ± 24.7	227.3 ± 52.3

*In A are shown the acoustic startle responses (measured by maximum voltage: vmax, mv) induced by stimuli of different intensities (from 80 to 100 dB). In B is shown the distance traveled (cm) in the circular corridor and in the novelty preference test (session 1). In C are shown the exploration (s) of the familiar (F) and the novel (N) objects and the discrimination index (%), in the object recognition test. In D are shown ACTH (pg/ml) and Corticosterone (CORT, ng/ml) levels in basal conditions. Means ± s.e.m. are shown*.

**Figure 7:**
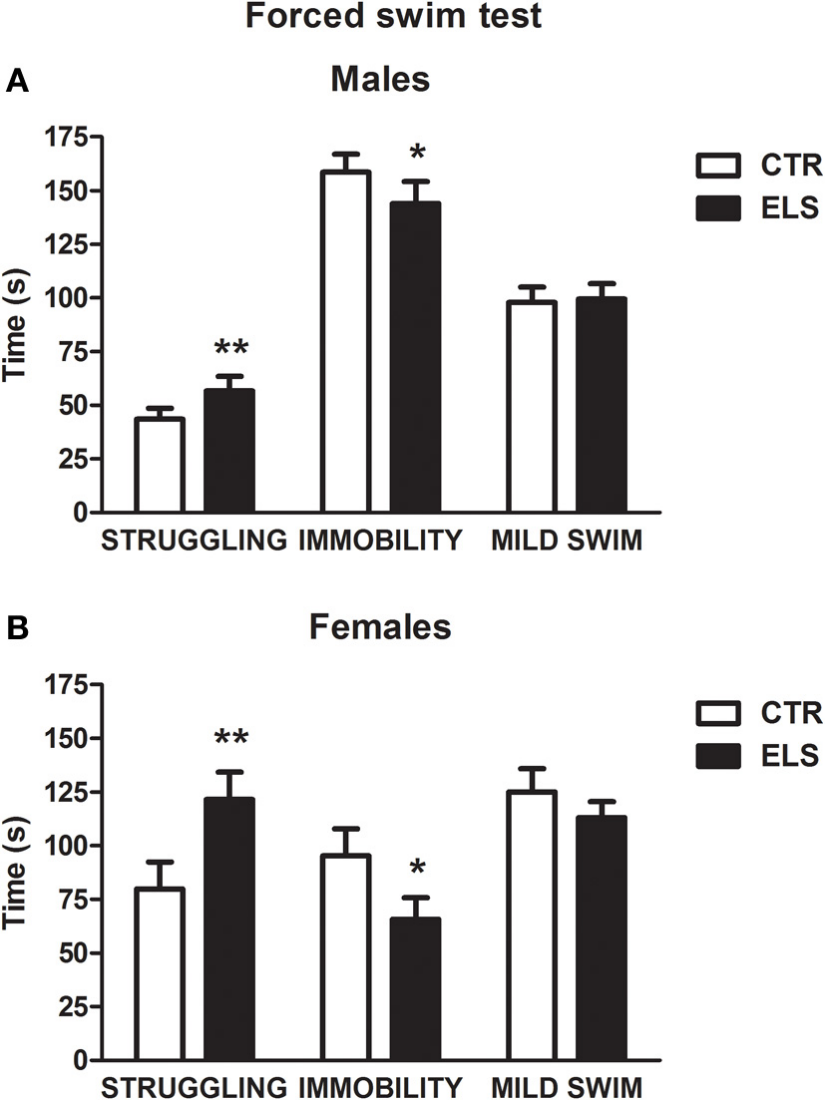
**Time in sec (X ± s.e.m.) in the forced swim test spent in struggling, immobility, and mild swim behavior, in males (A) and females (B), for control (CTR) and early-life stress (ELS) rats**. ^*^p < 0.05, ^**^p < 0.01 vs. CTRL. For struggling and immobility, GENDER and ELS effects were statistically significant, but not the interaction.

Regarding the forced swim behavior during the first 5 min of the test (Figure 7), ELS increased escape (struggling) and decreased immobility (floating) in both males and females, and the females were more active than the males. In contrast, mild swim behavior was not affected by the treatments. Regarding struggling, statistical analysis showed that the ELS [*F*_(1, 42)_ = 8.30, *p* < 0.01] and GENDER [*F*_(1, 42)_ = 27.51, *p* < 0.001] effects were significant; however, the interaction was NS. The statistical analysis of immobility behavior showed that ELS [*F*_(1, 42)_ = 4.59, *p* < 0.05] and GENDER [*F*_(1, 42)_ = 47.04, *p* < 0.001] were significant, with NS the interaction GENDER × ELS.

### Novelty-seeking: ELS increased in males the exploration of novel objects, but decreased the preference for novel spaces in both genders

The results regarding the distance traveled in the circular corridor test are shown in Table 3B. Statistical analysis showed that the GENDER factor was significant [*F*_(1, 48)_ = 8.03, *p* < 0.01]; however, the other factors and interactions were NS. Therefore, females were more active than males in an inescapable novelty situation, but ELS did not affect this measure.

**Figure 8:**
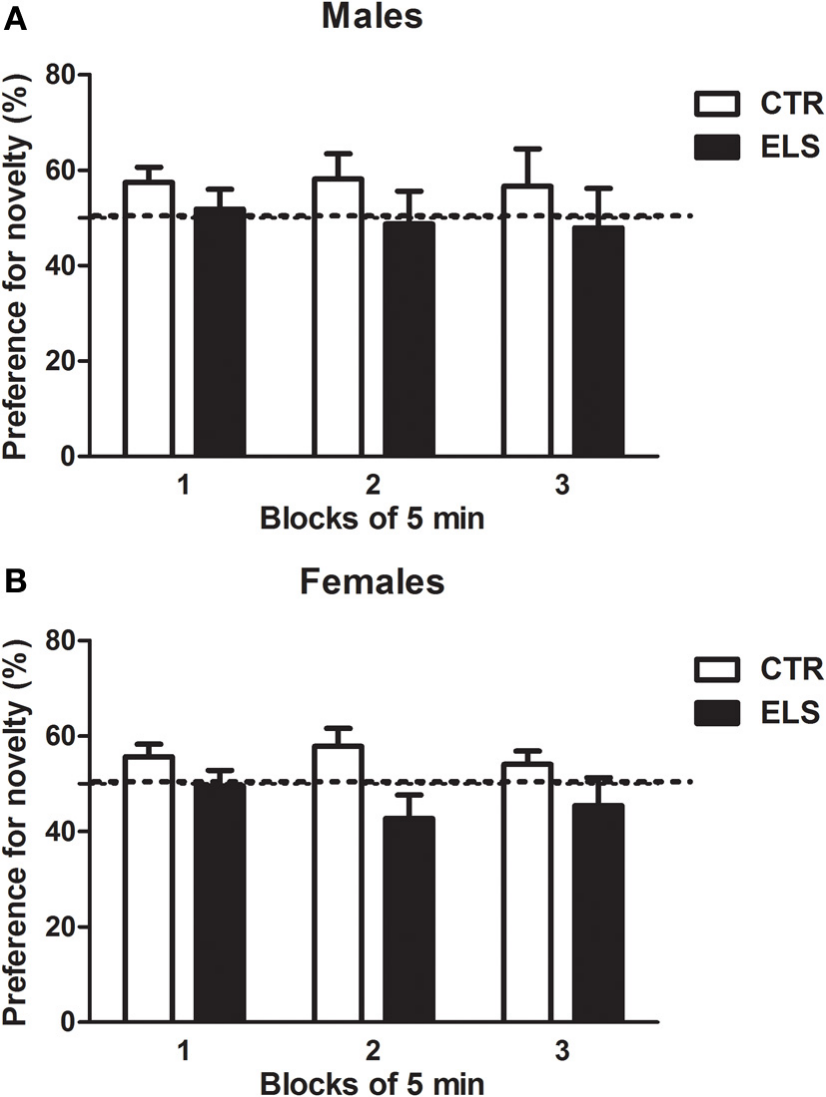
**Preference for a novel compartment (% of time), across three blocks of 5 min, in males (A) and females (B) for control (CTR) and early-life stress (ELS) rats**. The dotted line represents random preference (50 % of the time in each compartment). The ELS × COMPARTMENT effect was statistically significant. Only for CTR rats (regardless of gender) the preference for the novel compartment was higher than random (*p* < 0.05).

Later, the animals were exposed to another novel environment (one of two compartments of a CPP apparatus), and the distance traveled was measured. As Table 3B indicates, the females were again more active than the males [GENDER: *F*_(1, 45)_ = 6.73, *p* < 0.05]; however, ELS and GENDER × ELS were NS. After another session where the animals were confined to this compartment, the separation between both compartments was removed, and the preference for the novel environment was measured under free-choice conditions. To study the preference for the novel environment, the time spent in the novel compartment across 3 blocks of 5 min was compared to random preference (50%). As shown in Figure 8, ELS decreased the preference for novel spaces. The statistical analysis showed that ELS [χ 2 (1) = 4.18, *p* < 0.05] and ELS × COMPARTMENT [χ 2 (1) = 4.18, *p* < 0.05] were statistically significant; however, GENDER, COMPARTMENT, GENDER × ELS, GENDER × COMPARTMENT, ELS × GENDER × COMPARTMENT were NS. The decomposition of the interaction ELS × COMPARTMENT indicated that only in the control animals, the preference for the novel compartment was higher than the random preference (*p* < 0.05) regardless of gender.

When the subjects were exposed to 2 equal novel objects (A-A or B-B) and the time spent exploring the objects during the first 5 min was measured (Figure 6B), statistical analysis showed that ELS and GENDER were NS, but the interaction GENDER × ELS was significant [*F*_(1, 48)_ = 7.10, *p* = 0.01]. The decomposition of the interaction indicated that, in males, ELS increased the exploration of the novel objects [*F*_(1, 48)_ = 8.10, *p* < 0.01]. In the recognition memory test that was given the next day (with A and B objects present, novel and familiar), a repeated-measures analysis of variance was conducted with OBJECT as the within-subject factor and ELS and GENDER as the between-subject factors. As Table 3C indicates, as expected, all of the groups explored the novel object more than the familiar one [OBJECT:*F*_(1, 48)_ = 12.93, *p* = 0.001], whereas the other factors and interactions were NS. The discrimination index (Table 3C) analysis also indicated that ELS, GENDER and ELS × GENDER effects were NS, supporting the lack of differences in recognition memory.

### HPA axis. ELS decreased acth response to stress

**Figure 9:**
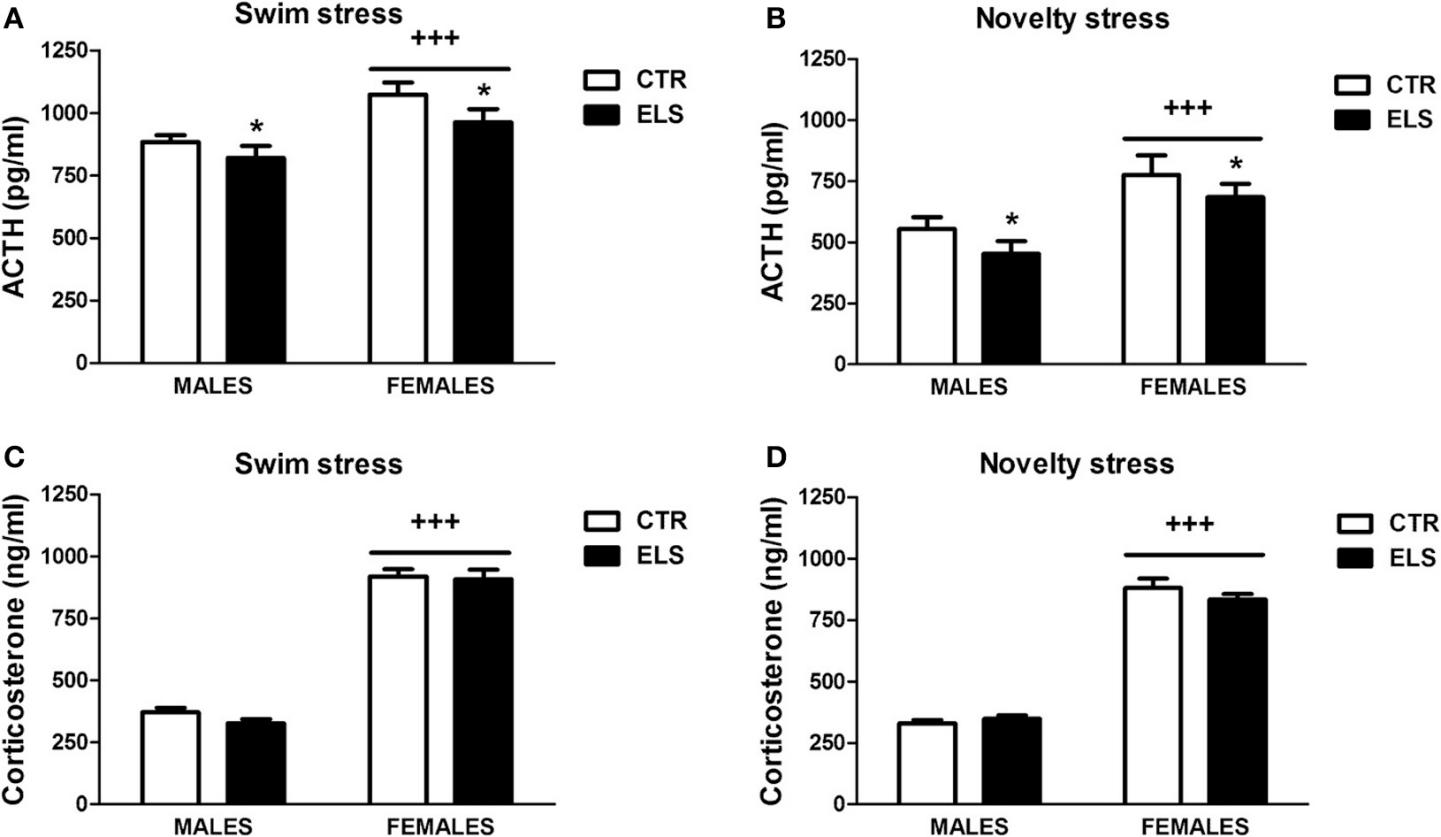
**ACTH (pg/ml) and corticosterone (ng/ml) levels (X ± s.e.m.) in response to swim stress (A,C) and novelty stress (B,D), in males and females for control (CTR) and early-life stress (ELS) rats**. ^*^p < 0.05 vs. CTR, ^+++^*p* < 0.001 vs. males. For ACTH, the ELS, GENDER, and STRESSOR effects were statistically significant, but not the interaction. For corticosterone, only GENDER was statistically significant.

The basal levels of ACTH and corticosterone were not affected by ELS (see Table 3D) and were higher in females than males [for ACTH: GENDER: *F*_(1, 42)_ = 6.39, *p* < 0.05; for corticosterone: GENDER: *F*_(1, 42)_ = 50.08, *p* < 0.001; ELS and GENDER × ELS were NS in both cases]. As the HPA axis reactivity to stress was analyzed after a novel environment (preference for novelty test) and after the FST, STRESSOR was included in the statistical analysis as a factor. The ACTH levels in response to the novel environment and to the FST were reduced by ELS (Figures 9A,B). In these tests, statistical analysis showed that: (1) ELS decreased the endocrine response to the tests in both males and females [ELS:*F*_(1, 90)_ = 6.07, *p* < 0.05]; (2) females had higher ACTH levels than males in response to the tests [GENDER: *F*_(1, 90)_ = 27.83, *p* < 0.001); and (3) ACTH levels in response to the FST were higher than in response to the novel environment [STRESSOR: *F*_(1, 90)_ = 73.26, *p* < 0.001]; however, the interactions between factors were NS. Regarding the corticosterone response to the tests (Figures 9C,D), GENDER was the only statistically significant factor [*F*_(1, 90)_ = 985.81, *p* < 0.001], indicating that females had higher corticosterone levels than males regardless of the stressor.

### Five choice serial reaction time task: ELS increased the number of errors made and perseverative responding in females

Several differences between groups were observed in the initial acquisition of the task. ELS slightly increased the number of days needed in phase F1 before moving to F2 [ELS: *F*_(1, 28)_ = 4.80, *p* < 0.05; GENDER and GENDER × ELS: NS; CTR-male: 4.5 ± 0.27 days; CTR-female: 4.5 ± 0.27 days; ELS-male: 5.8 ± 0.53 days, ELS-female: 4.9 ± 0.30 days]. The number of days needed in phase F5 to move to F6 were also increased by ELS [ELS: χ^2^(1) = 4.23, *p* < 0.05; GENDER and GENDER × ELS: NS; CTR-male: 12.3 ± 0.6 days; CTR-female: 11.6 ± 1.1 days; ELS-male: 13.9 ± 0.5 days, ELS-female: 15.0 ± 2.5 days].

**Figure 10:**
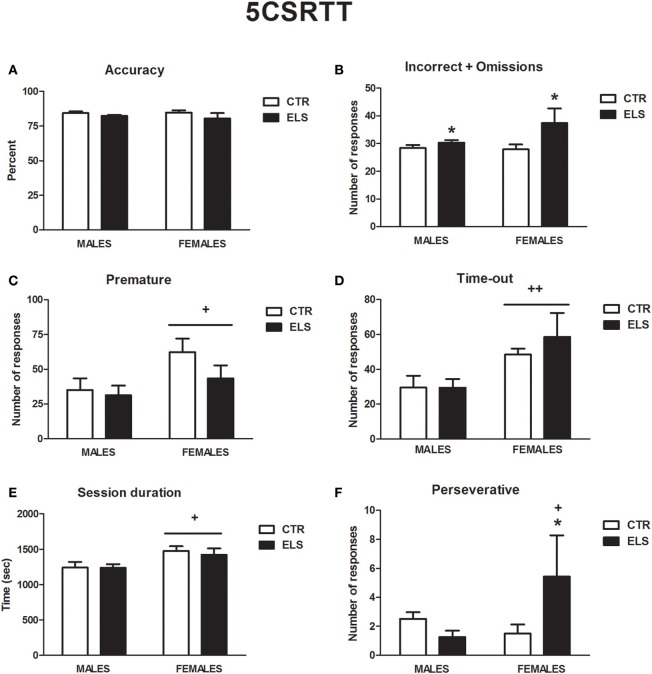
**Performance in the five choice serial time task (phase 6), in males and females for control (CTR) and early-life stress (ELS) rats**. ^*^p < 0.05 vs. CTR; ^+^*p* <0.05 and ^++^*p* <0.01 vs. males. For **(A)**, not effect was statistically significant. For **(B)**, the ELS effect was statistically significant. For **(C–E)**, the GENDER effect was statistically significant. For **(F)**, the GENDER × ELS effect was statistically significant.

All of the subjects were run to achieve the criteria needed for phase F6, but two rats from the ELS-female group never achieved the criteria needed to move to phase F7. The mean data from the last 3 days in F6 were analyzed as representative. The statistical analysis indicated that ELS did not affect the accuracy in the task (Figure 10A) or the correct responses (data not shown) but increased the total numbers of errors (incorrect+omissions) [Figure 10B, ELS: χ 2(1) = 4.44, *p* < 0.05, GENDER and GENDER × ELS: NS]. ELS also increased the number of perseverative responses in females (Figure 10F). In fact, one of the female ELS rats was excluded from the statistical analysis because it was an outlier due to the high number of perseverative responses. Even excluding this rat, ELS increased the perseverative response in females [GENDER × ELS: χ 2 (1) = 4.17, *p* < 0.05; GENDER and ELS: NS]. Other differences were only due to sex. The females needed more time to finish the session, regardless of ELS [Figure 10E, GENDER: *F*_(1, 28)_ = 7.98, *p* < 0.01; ELS and GENDER × ELS: NS], and had longer latencies when performing incorrect responses [data not shown, GENDER: *F*_(1, 28)_ = 5.45, *p* < 0.05; ELS and GENDER × ELS: NS]. The females also showed an impairment in inhibition as measured by an increase in premature responses [Figure 10C, GENDER: *F*_(1, 28)_ = 5.06, *p* < 0.05; ELS and GENDER × ELS: NS] and responses made during the TO [Figure 10D, GENDER: χ 2(1) = 9.68, *p* < 0.01; ELS and GENDER × ELS: NS]. In conclusion, ELS slowed the acquisition of the task, increased the number of errors made and increased perseverative response in females.

### Delay-discounting: ELS decreased cognitive impulsivity in females

**Figure 11:**
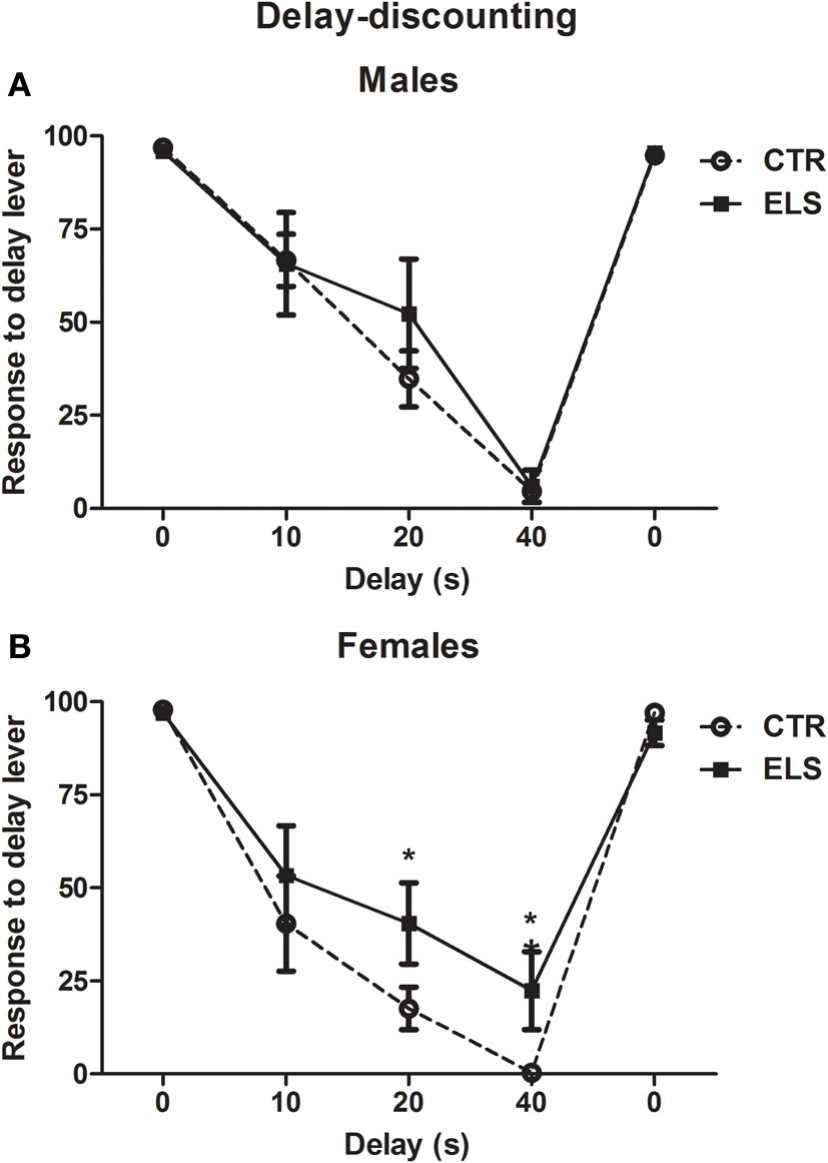
**Percent of the number of responses (X ± s.e.m.) to the “delay” lever with regard to the total number of responses in the delay-discounting task, with different delays between the response and the reward, in males (A) and females (B), for control (CTR) and early-life stress (ELS) rats**. ^*^p < 0.05 vs. CTR. The GENDER × ELS × DELAY effect was statistically significant.

During the pre-training sessions, no group differences were observed (data not shown). In Figure 11, the percentage of the number of responses to the delayed lever with regard to the total number of responses performed (excluding omissions) is shown (the average of the two last sessions in each delay). One rat from the ELS-male group was excluded because it stopped responding during all delays. As expected, at 0 s of delay, most of the animals preferred the “delayed” lever (4 pellets), and when the delay between the response and the reinforcement rats increased, the animals preferred the more immediate lever, although it only gave 1 pellet. At the end of the experiment, all of animals were again exposed to the delay 0 s condition, and the results showed that the animals preferred the “delayed” lever. The generalized linear model showed statistically significant effects for DELAY [χ^2^(4) = 1369.3, *p* < 0.001], ELS × DELAY [χ^2^(4) = 11.21, *p* < 0.05], GENDER × DELAY [χ^2^ (4) = 10.28, *p* < 0.01] and ELS × GENDER × DELAY [χ^2^(4) = 11.99, *p* < 0.01), but ELS, GENDER and ELS × GENDER were NS. To analyse the triple interaction, separate analyses were conducted for delay and gender. Regarding the gender differences, the results showed that females were more impulsive than males (less responses to the delayed lever) at 40 s of delay (*p* < 0.05), whereas differences between genders at 10 and 20 s of delay were only marginal (*p* = 0.055 and *p* = 0.052, respectively). ELS was statistically significant only for females at 20 and 40 s of delay (*p* < 0.05 in both cases). No differences in the other measures (latencies, omissions) were observed (data not shown). In conclusion, ELS only decreased impulsivity in females, and females were more impulsive than males.

## Discussion

The present model of ELS has was able to induce long-term effects in cognition, novelty-seeking, coping behavior and endocrine reactivity to stressors during adulthood, and several of the effects were gender-dependent. Unexpectedly, several of the effects were likely to be beneficial rather than detrimental, although negative consequences were also observed. The greater than expected beneficial (protective) effects may be related to a compensatory increase in maternal behavior, supporting the importance of measuring this behavior in any model of ELS.

### Maternal behavior

The distribution of maternal behavioral patterns in control biological mothers was similar to the patterns reported in Long-Evans (Champagne et al., 2003) and Sprague-Dawley (Dimitsantos et al., 2007) rats. Unexpectedly, ELS enhanced the active maternal behavior in biological mothers, increased the arched-back behavior and decreased the number of “off” nest episodes. This effect was dissipated when the bedding material was replaced.

These results are in contrast to the findings of Ivy et al. (2008), in which the restriction of nesting/bedding material impaired important aspects of maternal behavior. In this study, ELS increased the number of out of the nest episodes, which appears to be consistent with our casual observations that ELS enhanced the probability that several pups were out of the nest at the same time that the dam was in an arched-back posture. However, in Ivy et al. (2008), the treatment increased the number of episodes with dams “off” the nest and decreased occasional licking-grooming, clearly in contrast to the present study. Two primary differences exist between our treatment and the former study. We did not use a mesh bottom in the cage and did not provide the dam with an additional paper towel as a nesting material. Although these factors may be important, other unpublished data from our lab suggest that our strain/type of dams showed consistent compensatory maternal behavior during diverse types of ELS treatments, and compensatory maternal behavior may differ among strains (Millstein and Holmes, 2007). Furthermore, the capability to develop adequate maternal care in absence of nesting material varies profoundly between the strains of mice (Wei et al., 2010) and rats (Braw et al., 2009).

In the present study, caregiver (substitute) mothers did not display overt maltreatment toward the pups in contrast to previous data (Roth et al., 2009; Blaze et al., 2013). Under the conditions of the present study, the only putative sign of maltreatment observed was “stepping,” which decreased among the first seven post-natal days. Instead, “substitute” mothers displayed “normal” maternal care, such as licking-grooming. In the present work, the “substitute” mothers were “biological” mothers that participated in the study; however, in previous work, this methodological detail is not mentioned (Roth et al., 2009). Another important difference may be the use of primiparous vs. multiparous dams. Blaze et al. (2013) used multiparous dams, and the maternal behavior of primiparous and multiparous rats has important differences (Nephew et al., 2009). Potentially, in the present conditions, the combination of “normal” maternal care exhibited by biological and caregiver dams exert “compensatory” (“buffering”) effects that may account for several of the “beneficial” effects during adulthood. Treatments such as communal nesting (see Branchi et al., 2011 for a review) or post-natal handling (Liu et al., 1997) that increase maternal care have potent beneficial effects for pups in adulthood. In addition, a change in nursing behavior may also lead to variations in the nutritional/metabolic state of the pups, which exerts profound long-term consequences in behavior (see Lucassen et al., 2013 for a review). Future studies are needed to ascertain which of the potential factors (biological or substitute mother) is more relevant. Another question is to what extent the reported differences in maternal care received by male and female pups (male pups receive more licking-grooming, see Moore et al., 1997; Claessens et al., 2011) are maintained under conditions of adversity. Maternal care received by individual pups has not been measured in this study but may be involved in several of the gender-dependent effects observed during adulthood.

### Effects of ELS on pup behavior at adulthood

Baseline anxiety, as evaluated by the time spent in the open arms of an EPM, was not altered by ELS in either sex, although a non-significant trend to decrease open arm exploration was found. Another potential measure of anxiety, the amplitude of the ASR, was similarly not affected. Finally, the neuroendocrine response to stress (novel environment and forced swim) was significantly reduced rather than increased by ELS in the two sexes, suggesting reduced rather than enhanced emotional reactivity. This dissociation between HPA axis reactivity to stress and baseline anxiety has been previously observed in our laboratory (Márquez et al., 2006) and others (Courvoisier et al., 1996; Liebsch et al., 1998).

In the present study, we evaluated novelty-seeking behavior using different tests. Novelty-induced activity under inescapable conditions (circular corridor or CPP compartments) was not affected by ELS. However, the preference for novel spaces was decreased by ELS. In contrast, initial novel object exploration was increased by ELS but only in males. These data are in agreement with a study in squirrel monkeys that were subjected to early mild intermittent stress that preferred to interact with novel vs. familiar objects (Parker et al., 2007). The dissociation between the different measures of novelty-induced behavior has also been previously observed (Klebaur et al., 2001; Vidal-Infer et al., 2012).

The long-term object recognition (24 h) was not affected by ELS, with all groups exploring the novel object more than the familiar object. Most of the previous data with models of ELS, such as maternal separation/deprivation, evaluated only short-term memory (Aisa et al., 2007; Marco et al., 2013; Vivinetto et al., 2013), although a recent study (Garcia et al., 2013) reported a decrease in long-term memory in maternal separated animals.

Forced-swim behavior was also affected by ELS. The treatment increased, in both genders, active behaviors (struggling/climbing) and decreased passive behaviors (immobility/floating), which is suggestive of enhanced active coping strategies. In addition, ELS-exposed male and female rats showed a reduced endocrine (ACTH) response to the test. Because there is no evidence of a relationship between coping behavior in FST and the response of the HPA axis (Armario et al., 1995; Marti and Armario, 1996); the results are indicative of a beneficial effect of ELS on HPA responsiveness to stressors.

The present results regarding anxiety, coping behavior and HPA reactivity to stress are in contrast to previous studies reporting that different types of ELS induce anxiogenic-like effects in the EPM (Wigger and Neumann, 1999; Kalinichev et al., 2002; Brummelte et al., 2012). However, the EPM was not sensitive to ELS (Hulshof et al., 2011; Lajud et al., 2012), and the effects are dependent on the basal levels of the anxiety of the strain studied (Neumann et al., 2005). Regarding the FST, maternal separation increases active behavior (Rüedi-Bettschen et al., 2006), although other studies did not observe changes (Wang et al., 2011; Clarke et al., 2013) or describe increased passive coping strategies (Herpfer et al., 2012; Lajud et al., 2012; Martisova et al., 2012). Interestingly, the effects of ELS on the FST appear to be strain-dependent (Binder et al., 2011).

Although an important number of papers have reported that ELS in rats increased adult basal activity of the HPA axis and its reactivity to stress (Liu et al., 2000; Francis et al., 2002; Huot et al., 2002), there are also reports of decreased corticosterone response to stress in males (Greisen et al., 2005; Roman et al., 2006; Rüedi-Bettschen et al., 2006) and females (Rees et al., 2006). However, in several of these studies, ACTH was not measured or no differences in this hormone were found (Greisen et al., 2005; Rees et al., 2006; Roman et al., 2006; Rüedi-Bettschen et al., 2006). Given the well described dissociation between ACTH and corticosterone (see Armario, 2006; Bornstein et al., 2008, for reviews), an evaluation of the HPA axis reactivity should include both hormones. The decrease in ACTH reactivity to stress is in accordance with previous works in which increased maternal care was induced (i.e., post-natal handling or mothers that naturally display high levels of maternal care).

Animals exposed to ELS showed several differences in adulthood in impulsive-compulsive behavior, which were sex-dependent. Thus, ELS reduced cognitive impulsivity (impulsive choice), as evaluated in the delay-discounting paradigm, which cannot be explained by impaired motivation. The effect was found in females but not in males. In contrast to the decrease in cognitive impulsivity, ELS had no effect on impulsive action as measured by premature responding during the 5CSRTT. Impulsivity is a multidimensional construct, and the dissociation between impulsive action and impulsive choice has been repeatedly found after pharmacological treatments (Dellu-Hagedorn, 2006; Dalley et al., 2011). Moreover, impulsivity and compulsivity are mediated by partially overlapping but distinct neural circuits (Fineberg et al., 2010). In contrast, ELS increased compulsive-like behavior, as evaluated by the perseverative response in the 5CSRTT, but the effect was only observed in females. In addition, ELS increased the total number of errors in the 5CSRTT in both males and females, suggesting that the attention processes were also impaired. Cognitive dysfunction is also supported by the fact that ELS-exposed animals needed more days to reach the criteria needed to move on from particular phases of the task.

To our knowledge, the effects of ELS or altered developmental conditions on impulsive-compulsive behavior have been scarcely studied. Enhanced impulsive action (but no impulsive choice) has been previously reported after rearing pups in the absence of a mother (Lovic et al., 2011), supporting the dissociation between both traits. In contrast, several ELS treatments (maternal deprivation/separation, artificial rearing) decrease cognitive flexibility in both adult males (Oitzl et al., 2000; Enthoven et al., 2008; Wang et al., 2011; Baudin et al., 2012) and females (Lovic and Fleming, 2004; Mehta and Schmauss, 2011), as evaluated by other tasks.

### ELS-independent sex differences

Females were more active than males when forced to explore novel environments; however, no differences were observed in anxiety. These data are in accordance with the previous results from our laboratory (Peña et al., 2006, 2009) and others (Ray and Hansen, 2004; Brummelte et al., 2006; Llorente-Berzal et al., 2012). Moreover, the preference for novel spaces or the exploration of novel objects under conditions of free-choice was not sex-dimorphic in accordance with other studies (Cyrenne and Brown, 2011). Similarly, memory recognition was not sex-dependent, which is in agreement with previous data (Llorente-Berzal et al., 2012; Marco et al., 2013). Finally, FST data indicated that females struggled more than males and presented less immobility, which is in agreement with previous works (Alonso et al., 1991; Barros and Ferigolo, 1998; Brummelte et al., 2006; Simpson et al., 2012).

Regarding the HPA axis, the overall data indicate that Long-Evans markedly respond to relatively mild stressors, such as the exposure to a novel environment, but this result is not surprising because this strain is characterized by higher HPA activity (Tannahill et al., 1988; Bielajew et al., 2002). Nevertheless, we observed the well-described sex differences in rats: higher levels of ACTH and corticosterone during resting conditions associated with greater ACTH and corticosterone response to stressors in females (i.e., Hiroshige et al., 1973; Erskine et al., 1975; Atkinson and Waddell, 1997; Slotten et al., 2006; Iwasaki-Sekino et al., 2009; Brummelte et al., 2012).

In the 5CSRTT, females presented less inhibitory control than males, as evidenced by an increase in the premature responses (during the ITI) and during the TO. Premature responses have been considered as an index of impulsive action (Robbins, 2002). Gender differences in anticipatory responses are most likely dependent on the particular characteristics of the task used. When using a long ITI, recent data in 5CSRTT indicated that females had less inhibitory control than males (Burton and Fletcher, 2012). In contrast, in other conditions or in other versions of the task, the opposite results have been found (Jentsch and Taylor, 2003; Bayless et al., 2012). Regarding the delay-discounting test, females were also more impulsive than males under the present conditions. In other studies that used a delay-discounting procedure, females were also more impulsive than males in a particular rat strain that has low impulsivity (Perry et al., 2007). Therefore, both impulsive action and cognitive impulsivity are increased in females in comparison to males.

### ELS: vulnerability or resilience?

Until recent years, the dominant idea in Neuroscience was that ELS leads to detrimental effects at adulthood. The “cumulative” stress model proposes that the accumulation of stressors across the life span increases the risk for psychopathology in adulthood (McEwen, 1998). This hypothesis presents the same idea as the “two-hit” proposals (Choy et al., 2008, 2009); however, the present data add to other reports questioning whether ELS only leads to detrimental effects later in life. Central to this question is the extent to which an increase in maternal care induced by ELS may compensate or buffer the putative negative effects of stress.

Natural variations in maternal care have been elegantly associated with important long-term consequences in behavior and physiology by the group of Michael Meaney. The adult offspring of mothers that provide high maternal care to their litters showed less emotionality, enhanced hippocampal-dependent spatial learning/memory and higher sensorimotor gating-attention capabilities (see Zhang and Meaney, 2010 for a review). This behavioral profile is associated with a reduced plasma ACTH and corticosterone response to stress, enhanced hippocampal glucocorticoid receptor expression and reduced hypothalamic CRF expression (Liu et al., 1997). Although the importance of maternal care is widely recognized, the question remains as to whether the changes induced by ELS are related to changes in maternal care. Macrì and Würbel (2006) proposed that there are two independent mechanisms that mediate the long-term effects of ELS: the stressful component of the procedure for the pups and the changes in maternal care associated with the procedure. Perinatal low-moderate stressful situations may induce “protective” effects in adulthood; however, severe stressful situations result in detrimental effects (see Macrì et al., 2011 for a review). The stressful capability of our procedure is relatively mild, which may account for several of the protective effects that were found. The Macrì et al. (2011) proposal is similar to that of Parker et al. (2004), who demonstrated in non-human primates that moderate early stress “inoculates” (“immunizes”) the subject to better cope with future stressors, decreasing anxiety and HPA reactivity to stress (see for a review: Lyons et al., 2010; Parker and Maestripieri, 2011); being this “inoculation” dissociated from changes in maternal care (Parker et al., 2006).

Although dissociated, maternal care may be one of the critical mediators of the consequences of ELS. Importantly, the effects of ELS on maternal care were reported to vary among different strains (Millstein and Holmes, 2007). If such differences are strong, the same early life experimental manipulation may have markedly different consequences on maternal behavior and the long-term behavior of the pups, depending on the particular characteristics of the subjects/strains used. In several studies that observed an increase in maternal care induced by ELS, no negative or even beneficial effects have been described in adulthood (Marmendal et al., 2004; Llorente-Berzal et al., 2011; Own and Patel, 2013). This “buffering” effect of maternal care has also been described in humans, where high perceived parental care mitigates the negative consequences of ELS during adulthood (Kinnally et al., 2009). However, other data suggest that maternal care is apparently unable to compensate for the effects of ELS (Macrì et al., 2004; Neumann et al., 2005; Wei et al., 2010). In sum; the available data suggest that maternal care and ELS are dissociated; as shown in the present study, where a mild ELS was applied which in this case increased maternal care.

The final interpretation of the consequences of ELS as beneficial or detrimental must consider the environmental conditions during adulthood. Several researchers proposed that environmentally shaped developmental plasticity is a form of proper adaptation to the presumable environmental conditions that will be found during adulthood (Horton, 2005). Within this framework, Gluckman et al. (2007) suggested that a “mismatch” between the anticipated environment in early life and the actual adult environment increases the risk of pathology. Similarly, Boyce and Ellis (2005) proposed that ELS exerts both risk-augmenting and risk-protective effects, depending on the context where the adult subject is living. These “match-mismatch” models predict that after ELS, if the adult environment is also stressful, an increase in the coping behavior and adaptability to further exposures to stress will be shown (Champagne et al., 2009; Oitzl et al., 2010; Claessens et al., 2011; Schmidt, 2011). Within this model, natural variations in maternal care are strategies of success in different environments. Low licking-grooming offspring (adverse early life environment) is more “adapted” during adulthood under conditions of high stress (Barha et al., 2007; Champagne et al., 2008; Bagot et al., 2009). Moreover, maternal deprivation increases contextual and tone fear conditioning during adulthood (Oomen et al., 2010). Several results support the “cumulative” stress proposal, and others the “match-mismatch” hypothesis (Daskalakis et al., 2012). Recently, the “match-mismatch” model has incorporated the genetic vulnerability (Daskalakis et al., 2013), allowing the integration of recent data that suggest that ELS may be adaptive at adulthood as a function of the genetic background (Savignac et al., 2011; van der Doelen et al., 2013). However, maternal behavior has not been measured in all of these studies (Oomen et al., 2010; Savignac et al., 2011; van der Doelen et al., 2013), and further data is needed to integrate the relative contributions of the mentioned factors (maternal behavior, intensity of the ELS and adult context) to shape vulnerability vs. resistance to stress.

In the present study, animals were exposed to a mild stressor in early life, and within this theoretical framework, they may be more “adapted” in adulthood under conditions of mild stress. In agreement with this model, no “detrimental” effects were observed with low-intensity stressors (EPM, circular corridor, preference for novel spaces), and “protective” effects were observed with higher intensity stressors (decrease in ACTH response to forced swim and increase in active behaviors in that test). However, in other contexts (delay-discounting or 5CSRTT procedures), no available data regarding the HPA response to those tests to evaluate the intensity of the stress induced were observed. Moreover, the attribution of “protective” or “detrimental” properties to a given phenotype may be a matter of debate. For example, immobility in the forced swim is generally assumed to reflect a failure in coping, but other authors have suggested that this finding may reflect a relatively successful coping strategy that employs energy conserving behaviors (see Raftogianni et al., 2012 for a discussion).

In conclusion, the early treatment used in the present study induced a compensatory increase in maternal care. Long-term effects were observed during adulthood, some of which were gender-dependent and were dependent on the behavioral/cognitive domain evaluated. Thus, males and females are differentially affected by ELS; however, overall, neither gender is more vulnerable. Several of the consequences appear to be “negative,” such as a decrease in attention and an increase in compulsive-like behavior in the 5CSRTT. However, other consequences are “protective,” such as a decrease in HPA reactivity to stressors, an increase in active coping strategies in the FST or a decrease in cognitive impulsivity in a delay-discounting procedure. Although the present model may induce only a mild level of stress and different results may be obtained with higher intensities, the present data suggest that under several circumstances, ELS may be adaptive in promoting resilience to further challenges.

### Conflict of interest statement

Roser Nadal received fees from Panlab, SLU during 2009–2012. The company has no role in the design or writing of the MS. The other authors declare that the research was conducted in the absence of any commercial or financial relationships that could be construed as a potential conflict of interest
